# Spontaneous pneumomediastinum as the only CT finding in an asymptomatic adolescent positive for COVID-19

**DOI:** 10.1259/bjrcr.20200051

**Published:** 2020-05-15

**Authors:** Davide Bellini, Miriam Lichtner, Simone Vicini, Marco Rengo, Cesare Ambrogi, Iacopo Carbone

**Affiliations:** 1 Department of Radiological Sciences, Oncology and Pathology, "Sapienza" University of Rome, ICOT Hospital, Via Franco Faggiana 34, 04100, Latina, Italy; 2 Department of Diagnostic and Interventional Radiology, Santa Maria Goretti Hospital, Via Canova, 04100, Latina, Italy; 3 Infectious Diseases Unit, "Sapienza" University of Rome, Santa Maria Goretti Hospital, Via Canova, 04100, Latina, Italy

## Abstract

The typical findings on CT in patients affected by novel COVID-19 (coronavirus disease 2019) pneumonia are characterized by ground-glass opacities and/or air space consolidation mainly bilateral and peripherical in distribution, including crazy paving pattern and reversed halo sign. We hereby describe a case of an adolescent male tested positive for COVID-19 with mild respiratory symptoms and presenting with pneumomediastinum as the only CT finding.

## Case presentation

A 17-year-old young male was admitted to our Emergency Department complaining mild dyspnoea as the only clinical symptom. He experienced respiratory tract infection symptoms that lasted 2–3 days about 2 weeks before hospital admission, mainly represented by cough, sore throat, nasal congestion, malaise and temperature between 37 and 38°C. No specific pharmacological treatment but paracetamol was administered in that occasion. No other significant clinical or anamnestic data were reported.

On physical examination, heart rate was 60 beats per minute, blood pressure 120/70 mmHg, respiratory rate 24 breaths per minute and oxygen saturation 96–97% while he was breathing ambient air. His oropharynx was clear and without erythema or exudates. The neck was supple, without palpable abnormalities. The lungs were clear on auscultation.

His parents had been hospitalized to our COVID-19 Hospital with respiratory symptoms 1 week before, both affected by COVID-19-associated interstitial pneumonia, documented by multidetector CT (MDCT). Due to his close contact with positive subjects for a long period (they share the same apartment) and the mild respiratory symptoms, the patient was managed as a potential COVID-19 infection case.

## Investigations and image findings

Full blood count, inflammatory markers and chest MDCT were ordered. Laboratory tests revealed normal red blood cell count (hemoglobin 15.7 g dl^−1^, hematocrit 45.5%, mean corpuscular volume 81 fl), a slightly increased white cell count (11640/mm3, with 76.3% neutrophils, 16.7% lymphocytes, 5.8% monocytes and 0.9% eosinophils), normal platelet count and C-reactive protein (CRP) level (0.14 mg dl^−1^, normal 0–0.5). The results of coagulation tests were normal.

The nasopharyngeal swab performed at the admission turned out positive for SARS-CoV-2 with a real-time polymerase chain reaction assay indicating a low viral replication, such as for resolution phase of infection.

**Figure 1.**
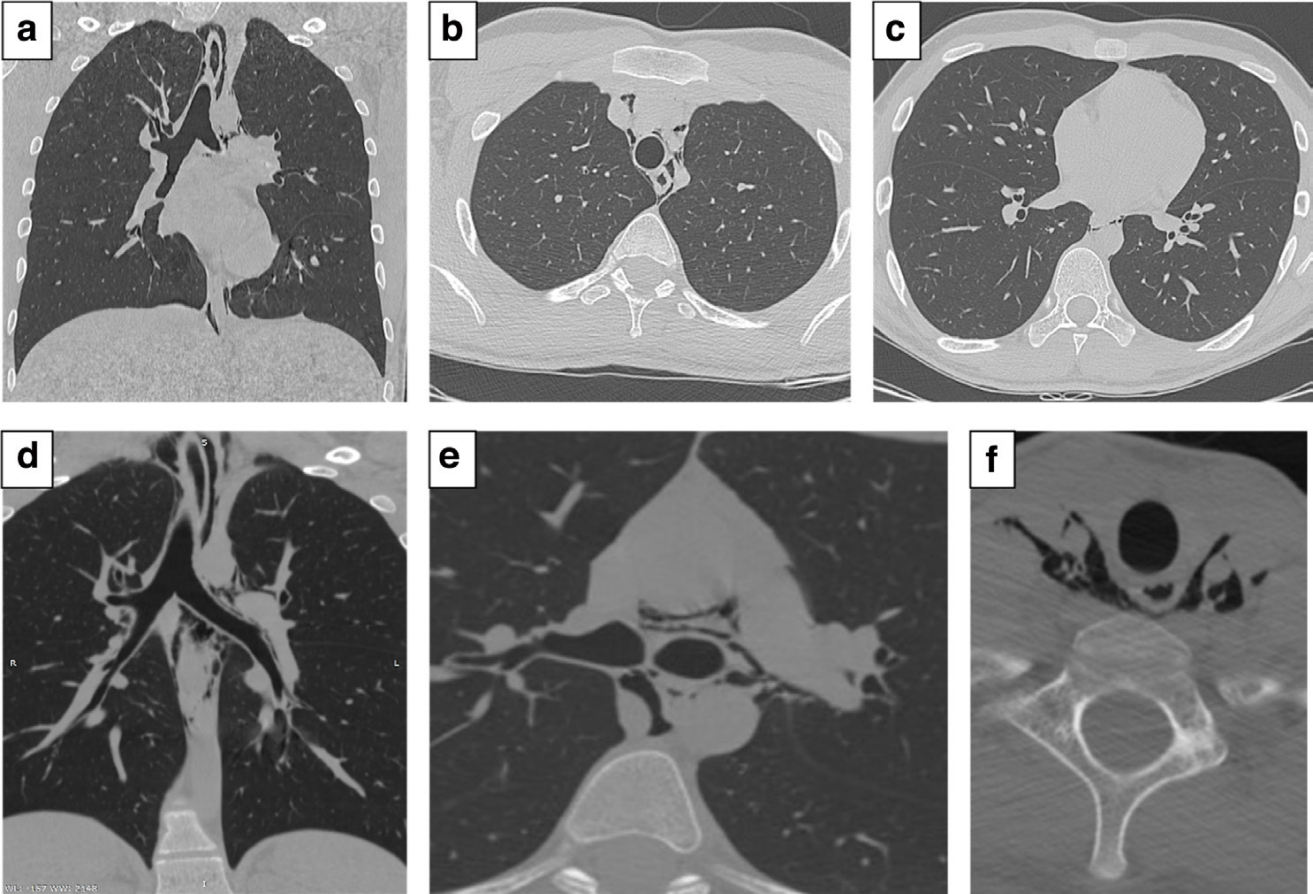
Coronal (A) and axial chest (B upper lobes; C, lower lobes) MDCT images showing normal lungs. The lungs appear dark gray and no abnormalities are seen throughout the entire parenchyma, with no evidence of pulmonary lesions along bronchovascular bundles and subpleural areas. No MDCT findings of pulmonary emphysema are seen either. Coronal (D) and axial chest (E) and neck (F) MDCT images demonstrating pneumomediastinum extending from the gastroesophageal junction up towards the neck. The coronal reconstruction (D) shows the accumulation of a discrete amount of free air within mediastinal space, surrounding the trachea and esophagus and reaching the thoracic outlet. The axial view at the level of the carina (E) demonstrates air pockets extending between the two main bronchi, aortic arch and descending thoracic aorta. The axial view at the level of infrahyoid region (F) shows the emphysematous involvement occurring in parapharyngeal, carotid and paratracheal neck spaces.

MDCT scan of the lungs was unremarkable: no typical, atypical or indeterminate parenchymal alterations were detected. Surprisingly, a discrete amount of pneumomediastinum extending up towards the neck was noted (Figure 1, Supplementary Video 1)

Supplemental Material 1

## Outcome and follow-up

Since the particular clinical scenario, the patient was referred to our Infectious Disease Department for conservative treatment, further evaluation, and monitoring evolution of such life-threatening condition.

## Discussion

Since the first report of novel pneumonia (COVID-19) in Wuhan, China,^1^ and its rapid spread throughout Europe, especially in Italy, there has been considerable discussion concerning different patterns of presentation at MDCT examination, aimed at improving diagnostic accuracy in order to compensate for the high percentage of false-negative results of real-time polymerase chain reaction assay.^2,3^

So far, the representative CT manifestations of COVID-19 are considered bilateral ground-glass opacities with or without consolidation involving posterior and peripheral lungs, often characterized by crazy paving pattern and reversed halo sign.^4,5^

However, with further analysis of various cases being published, a wide spectrum of CT imaging features is emerging everyday into routine clinical practice. Moreover, an important clinicoradiological dissociation has been highlighted in many of COVID-19 cases.^6^ Therefore, radiologists and all medical practitioners dealing with these patients should be aware of all new possible presentations of COVID-19 in order to prevent diagnostic errors and clinical underestimation, particularly in case of asymptomatic or paucisymptomatic individuals.

Spontaneous pneumomediastinum typically occurs due to alveolar rupture when a significant increase in intrathoracic pressure arises, as seen in case of coughing, sneezing or vomiting. Our patient experienced cough lasting for 2–3 days about 2 weeks before admission and one delayed symptom, mild dyspnoea. Two different hypotheses could explain pneumomediastinum in this case: first, intensive cough and subsequent pneumomediastinum occurred at the time of respiratory symptoms and still visible after 2 weeks. Second, spontaneous alveolar septa rupture as sequelae of diffuse interstitial-alveolar damage induced by COVID-19, not visible at our CT exam, that weakened lung parenchyma increasing risks of alveolar rupture. The later hypothesis is also corroborated by recent evidences that reported diffuse interstitial-alveolar inflammatory infiltrates as the main histopathological change associated with COVID-19.^7^

At this point of pandemic diffusion of the COVID-19 infection, due to the rapid spread of the disease worldwide and the lack of universally shared guidelines regarding common indications for thoracic imaging in patients with COVID-19 pneumonia [when to perform CT, how many times during the course of illness and at which purpose (*e.g*. screening, monitoring evolution, assessing possible complications)], an increasing number of different radiological presentations should be considered and pneumomediastinum is one of them, as it could represent a life-threatening event. As far as we know, this is the first case of COVID-19 infection complicated with pneumomediastinum in the absence of detectable alterations of lung parenchyma.

## Learning objectives

1. Different chest CT imaging manifestations are observed in patients with COVID-19. The case presented describes pneumomediastinum as the only and unexpected CT finding in an asymptomatic patient tested positive for COVID-19. No similar cases have been reported in the literature so far.
2. Pneumomediastinum has to be considered in case of COVID-19 infection and requires urgent attention because it could be a life-threatening complication.
3. Radiologists and all medical practitioners dealing with patients affected by COVID-19 should be aware of all new possible presentations in order to prevent diagnostic errors and clinical underestimation, particularly in case of asymptomatic or paucisymptomatic individuals.
